# YsxC, an essential protein in *Staphylococcus aureus *crucial for ribosome assembly/stability

**DOI:** 10.1186/1471-2180-9-266

**Published:** 2009-12-18

**Authors:** Elizabeth L Cooper, Jorge García-Lara, Simon J Foster

**Affiliations:** 1 Department of Molecular Biology and Microbiology, University of Sheffield, Sheffield S10 2TN, UK

## Abstract

**Background:**

Bacterial growth and division requires a core set of essential proteins, several of which are still of unknown function. They are also attractive targets for the development of new antibiotics. YsxC is a member of a family of GTPases highly conserved across eubacteria with a possible ribosome associated function.

**Results:**

Here, we demonstrate by the creation of a conditional lethal mutant that *ysxC *is apparently essential for growth in *S. aureus*. To begin to elucidate YsxC function, a translational fusion of YsxC to the CBP-ProteinA tag in the staphylococcal chromosome was made, enabling Tandem Affinity Purification (TAP) of YsxC-interacting partners. These included the ribosomal proteins S2, S10 and L17, as well as the ^β^' subunit of the RNA polymerase. YsxC was then shown to copurify with ribosomes as an accessory protein specifically localizing to the 50 S subunit. YsxC depletion led to a decrease in the presence of mature ribosomes, indicating a role in ribosome assembly and/or stability in *S. aureus*.

**Conclusions:**

In this study we demonstrate that YsxC of *S. aureus *localizes to the ribosomes, is crucial for ribosomal stability and is apparently essential for the life of *S. aureus*.

## Background

*Staphylococcus aureus *colonises the nares and skin of approximately one-third of the healthy global population [1] and is responsible for a wide variety of infections both in hospitals and the community [2-4]. The increasing antibiotic resistance of *S. aureus *has led to the search for alternative drug targets. Amongst them, proteins indispensable for cellular viability are optimal candidates. There are currently about 15 essential proteins from bacterial genomes used as antibiotic targets encompassing a restricted set of microbial processes, including DNA replication and repair, fatty acid and protein biosynthesis, and cell wall synthesis [5]. A large number of essential proteins remain to be investigated for novel antimicrobial development.

In a genome-wide study in *Bacillus subtilis *the IPTG-inducible P_spac _conditional expression system was used to determine gene essentiality [6]. A subset of 15 genes identified in this screening had no significant homology to any gene of known function, and included the well-conserved Era/Obg family of GTP binding proteins [6]. The latter belongs to a diverse superfamily of the often referred to as low molecular weight GTPases, which act as molecular switches in the regulation of crucial cellular processes across all domains of life, including: intracellular and membrane signalling, vesicular transport, cell division, chromosome partitioning, protein targeting and ribosomal function [7].

Although very few of the bacterial low molecular weight GTPases have well characterised roles, there is increasing evidence that members of the Era/Obg family of GTPases are involved in ribosome function, assembly or stability. Work on Era, Obg, YjeQ/YloQ, YlqF, YphC, and YsxC in *E. coli *and *B. subtilis *has indicated associations of these proteins with ribosomal subunits and changes in ribosomal profiles [8-10]. Ribosome profiles, created by separation of ribosome constituents on a sucrose gradient, show a decrease in whole 70 S ribosomes with an concomitant increase in 30 S and 50 S ribosomal subunits after depletion of the protein of interest [9,11-15].

YsxC in *B. subtilis *(YihA in *E. coli*) is an ortholog of the Era/Obg family of GTP-binding protein that has been reported to be essential in *B. subtilis*, *E. coli, S. pneumoniae*, *H. influenzae*, and *M. genitalium *[9,16,17]. We have previously solved the crystal structure of the *B. subtilis *YsxC in its open and closed conformations, proven its ability to complex with GDP and GTP, and shown the conformational changes occurring upon nucleotide binding and GTP hydrolysis [18].

A *B. subtilis *mutant with *ysxC *under the control of the regulatable P_spank _promoter has revealed that depletion of the protein led to the accumulation of intermediate 50 S subunits (described as 44.5 S subunits) different from those seen upon depletion of similar GTPases YphC and YlqF [9]. However, as with YlqF and YphC depletion, intermediates lacked ribosomal proteins L16, L36 and possibly L27. Other putative ribosomal interacting partners of YsxC have been suggested by Wicker-Planquart and co-authors [10]. YsxC is likely to be essential across eubacteria.

In this study we demonstrate that YsxC of *S. aureus *localizes to the ribosomes, is crucial for ribosomal stability and is essential for the life of *S. aureus*.

## Results

### YsxC is essential in *S. aureus*

**Figure 1:**
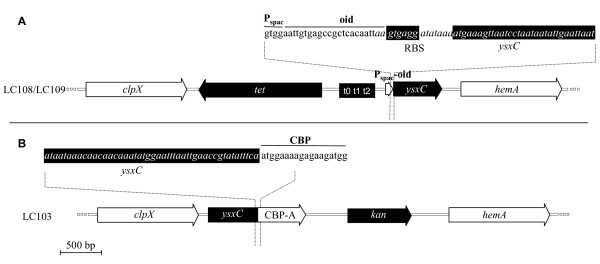
**Detailed scale representation of the P _spac_~*ysxC *(LC108/LC109) and *ysxC*::TAP-tag (LC103) chromosomal constructs**. λ_red _recombination allowed highly specific chimera construction resulting in the Tet-T-P_spac _or TAP-tag-kan cassette insertions. The relevant sequence junctions are shown for both constructs. Chromosomal sequence is shown in italics and relevant features generated by λ_red _recombination are underlined.

**Figure 2:**
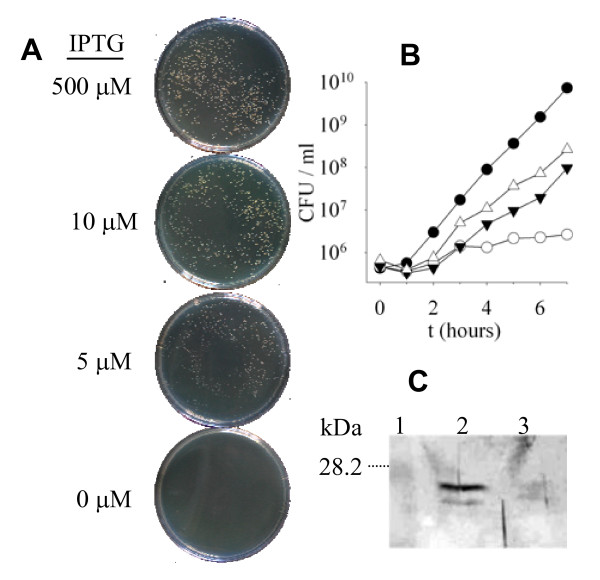
**YsxC requirement for *S. aureus *growth**. A) Strain LC109 (SH1000 P_spac_~*ysxC*/pGL485) was grown on BHI agar plates containing 20 μg ml^-1 ^Cam and 500 μM, 10 μM, 5 μM or 0 μM IPTG overnight. B) Exponentially growing cultures of strains SH1000 (●) and LC109 (SH1000 P_spac_~*ysxC*/pGL485) (○,τ,ρ) were washed and resubcultured to approximately 1×10^6 ^CFU ml^-1 ^in BHI (●) or in BHI supplemented with 20 μg ml^-1 ^Cam plus different concentration of IPTG: 0 (○), 10 μM (▼) or 1 mM (△). Growth was monitored as CFU/ml. c) Western blot using anti-YsxC polyclonal antibodies. Strains SH1000 and LC109 (SH1000 P_spac_~*ysxC*/pGL485) were grown to an OD_600 _= 0.5 in BHI and BHI plus 20 μg ml^-1 ^Cam, respectively. Cells were harvested by centrifugation, the membrane protein fraction extracted and samples were separated by 12% (w/v) SDS-PAGE. Lane: 1, the size of molecular weight markers separated on the same gel is indicated; 2, SH1000; 3, LC109.

To test whether *ysxC *was essential in *S. aureus*, a strain containing a single chromosomal copy of *ysxC *under the control of a regulatable promoter (P_spac_), repressed by LacI and requiring the inducer IPTG for expression was constructed as indicated in Material and Methods (See also Figure 1). Growth of LC109 (SH1000 P_spac_~*ysxC*/pGL485) at several IPTG concentrations (0 μM, 5 μM, 10 μM and 500 μM) was analysed on BHI agar plates supplemented with chloramphenicol to ensure maintenance of the *lacI*-containing plasmid (Figure 2A). Strong growth can be seen on the plate containing 500 μM IPTG with distinctive single colonies, which are absent on the plate without IPTG. The phenotype on solid medium was further confirmed in liquid medium (Figure 2B). In a different experiment it was shown that the presence or absence of IPTG does not affect growth of the wild type SH1000 strain (data not shown), while growth of LC109 (SH1000 P_spac_~*ysxC*/pGL485) is IPTG concentration dependent (Figure 2B). No distinguishable alterations were observed on YsxC-depleted cells under light or transmission electron microscopy (data not shown). The number of viable counts on LC109 incubated in the absence of IPTG remained virtually unchanged, while in the presence of 1 mM IPTG it increased by 2 logs. Interestingly, even at 1 mM IPTG, LC109 (SH1000 P_spac_~*ysxC*/pGL485) had a growth defect when compared to the wild type SH1000 strain (2.8×10^8 ^and 7.3×10^9 ^CFU after 7 h, respectively). These results demonstrate that *ysxC *is apparently essential for growth of *S. aureus *in these conditions.

To study if the reduction in growth rate seen using the *ysxC *conditional lethal strain LC109 (SH1000 P_spac_~*ysxC*/pGL485) correlated with a concomitant depletion of YsxC, protein levels after growth without IPTG were analysed. As indicated above, cells showed a severe growth defect when IPTG was lacking, thus limiting the yield for biochemical analysis. To overcome this, a higher initial inoculum (OD_600 _= 0.01) was used and cultures were grown with choramphenicol and IPTG (with 500 μM or without). At this inoculum density, without IPTG the growth rate of LC109 (SH1000 P_spac_~*ysxC*/pGL485) was still approximately 1 log below that of SH1000 after 5 hours of growth (data not shown). Equal amounts of material purified by ultracentrifugation were analysed by SDS-PAGE (data not shown) and Western blotting, probing with anti-YsxC polyclonal antibody (See Methods; Figure 2C). In SH1000 there is a major YsxC cross-reactive band of ~26 kD and a minor band of ~25 kD, corresponding to a size similar to the predicted molecular weight, i.e., 23 kD. Both bands show lower intensity in LC109 (SH1000 P_spac_~*ysxC*/pGL485) grown without IPTG. Hence, *ysxC *downregulation is accompanied by a decrease in YsxC concentration in the cell.

### Purification of YsxC interacting partners

**Figure 3:**
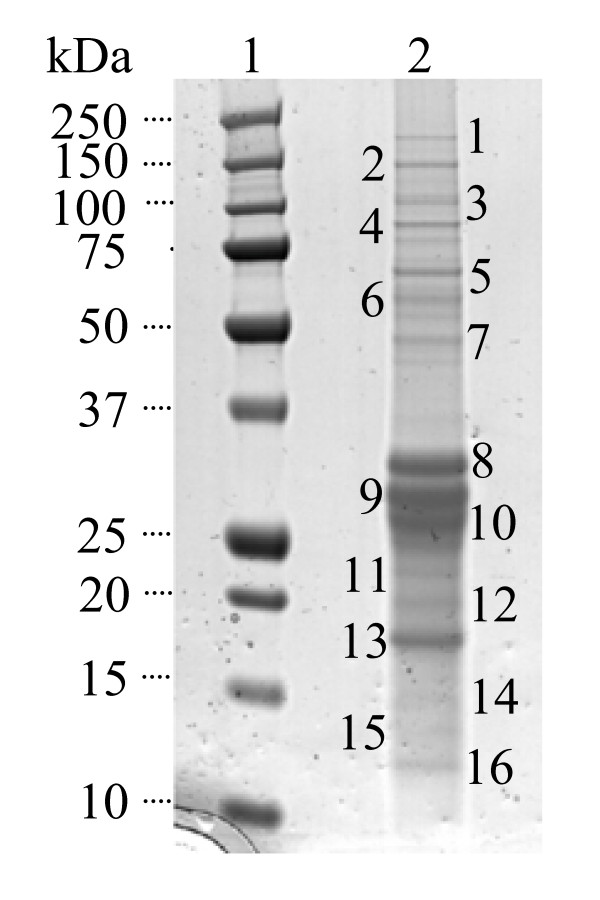
**Identification of YsxC interacting proteins**. Proteins were separated on a 4-12% (w/v) SDS-PAGE gradient gel and silver stained. Lane: 1, molecular mass markers of sizes shown; 2, YsxC complex proteins from 15 l of original culture. The band numbers correspond to those that were analysed by mass spectrometry.

One method used to elucidate the function of a protein of interest is to search for protein partners with which it interacts in the cell. In order to identify proteins interacting with YsxC, the protein was TAP-tagged [strain LC103 (SH1000 *spa*::*tet ysxC*::TAP)] and an interactive complex purified as described in Materials and Methods. The resulting proteins were separated by SDS PAGE and silver stained (Figure 3). 16 distinctive protein bands found in the eluted YsxC complex were trypsin digested and the amino acid sequence of the resulting fragments determined by mass spectrometry. Subsequently, a MASCOT search for proteins in the database containing these sequences was carried out. Table 1 shows the most probable identity of each of the bands as per its Mowse score. 10 of the 16 bands were identified as proteins from *S. aureus*, one band was not identified, and four of them (casein and keratin) corresponded to preparation contaminants.

**Table 1:** MASCOT search results for YsxC partners

Band no.	Gene name	Protein	Mowse score (threshold level) *	No. of fragments
1	not identified			
2	*rpoC*	DNA-directed RNA polymerase beta' chain	40 (36)	2
3	*secA*	preprotein translocase secA subunit	20 (35)	1
4	*pflB*	formate acetyltransferase	27 (36)	1
5	*glpD*	aerobic glycerol-3-phosphate dehydrogenase	167 (37)	6
6	Casein			
7	*tuf*	putative elongation factor Tu	39 (36)	1
8	*rpsB*	30 S ribosomal protein S2	121 (36)	3
9	*ysxC*	putative GTP-binding protein	227 (36)	2
10	Keratin			
11	Casein			
12	Casein			
13	*rpsE*	30 S ribosomal protein S5	246 (38)	5
14	Casein			
15	*rplQ*	50 S ribosomal protein L17	98 (41)	3
16	*rpsJ*	30 S ribosomal protein S10	173 (39)	3

^1 ^Mowse score = -10*Log(P), where P is the probability that the observed match is a random event. The higher the number, the better the match. Individual ions with scores greater than the threshold level (in brackets) indicates identity or extensive homology (p < 0.05).

The band that had the highest probability of a match was Band 13. Its Mowse score for 30 S ribosomal protein S5 was 246 with a threshold level of 38. Since 5 fragments from this band matched to this protein the identification is highly probable. Other bands with high match identities were Band 5 (aerobic glycerol-3-phosphate dehydrogenase), Band 8 (30 S ribosomal protein S2), Band 15 (50 S ribosomal protein L17) and Band 16 (30 S ribosomal protein S10) (Table 1). YsxC, the protein originally tagged, was also identified as a high match band (Band 9, 227(36)). All these proteins matched at least 2 fragments from the band. For 2 parent ions with a score of 95% or better, one can assume that the proteins has been identified.

Other interacting bands identified with a score indicative of extensive homology (i.e., 36, See Methods) were bands 2 and 7, and corresponded to the DNA-directed RNA polymerase beta' chain protein and putative elongation factor Tu. However, although the former matched 2 fragments, the latter, like SecA and PflB, were one hit matches, which would require further validation to be considered as legitimate YsxC partners. Similarly, Bands 3 and 4 corresponded to casein, a protein not present in *S. aureus *but a common preparation contaminant.

TAP tagging has not previously been reported in *S. aureus *therefore it was important to eliminate the possibility that any of the proteins identified, corresponded to purification artefacts. An independent purification of an unrelated TAP-tagged protein of *S. aureus *most likely participating in phospholipid metabolism and also purifying with the membrane fraction was carried out (YneS/PlsY; García-Lara and Foster, unpublished). It revealed interactions with proteins also encountered in our search for YsxC partners: 30 S ribosomal protein S5, elongation factor Tu and aerobic glycerol-3-phosphate dehydrogenase (data not shown). Although these data do not exclude the corresponding proteins as legitimate interacting partners of YsxC and YneS/PlsY, the involvement of these two proteins in different aspects of bacterial physiology suggests the common partners as likely artefacts of the purification procedure. Overall, the protein partners resulting from our experiments suggest YsxC as a ribosome-interacting protein.

### Subcellular localisation of YsxC

**Figure 4:**
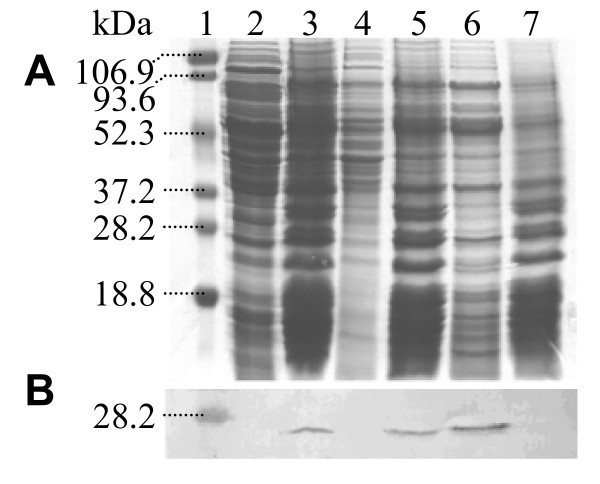
**Subcellular localisation of YsxC**. The ribosome-containing fraction of *S. aureus *SH1000 was made by ultracentrifugation after cell breakage and removal of cellular debris. Lane: 1, pre-stained molecular mass markers; 2, supernatant after ultracentrifugation; 3, pellet resuspended in buffer, containing 0.5% (v/v) Triton X-100, equal to that of the original suspension; 4, supernatant after the ultracentrifugation step was repeated; 5, pellet resuspended in buffer containing 1 M ammonium chloride (NH_4_Cl); 6, supernatant after further ultracentrifugation; 7, pellet resuspended in an equal amount of buffer containing 1 M NH_4_Cl. Samples were resolved by 12% (w/v) SDS-PAGE and A) Coomassie Blue stained, or B) Western blotted with antibodies against YsxC. Each lane contains the equivalent of 1 ml of original culture.

The TAP tagging experiment identified several ribosome-associated proteins as YsxC interacting partners. To examine the putative association of YsxC with ribosomes, a co-purification experiment was carried out. Staphylococcal ribosomes were extracted from other cellular materials by several ultracentrifugation and washing steps, and core ribosomes were depleted of accessory ribosomal proteins by ammonium chloride extraction. Equivalent samples from different stages of the purification process were separated by SDS-PAGE, Western blotted and immuno-detected with anti-YsxC antibodies (Figure 4). YsxC is in the insoluble fraction following the initial ultracentrifugation of a total cell extract (lane 3) and remains in the insoluble fraction after solubilisation of the membranes with Triton X-100 (lane 5). When this insoluble fraction was resuspended in 1 M NH_4_Cl, YsxC was solubilised (lane 6). These results suggest that YsxC is associated with the ribosome but is not a core ribosomal protein.

### Association of YsxC with specific ribosomal subunits

**Figure 5:**
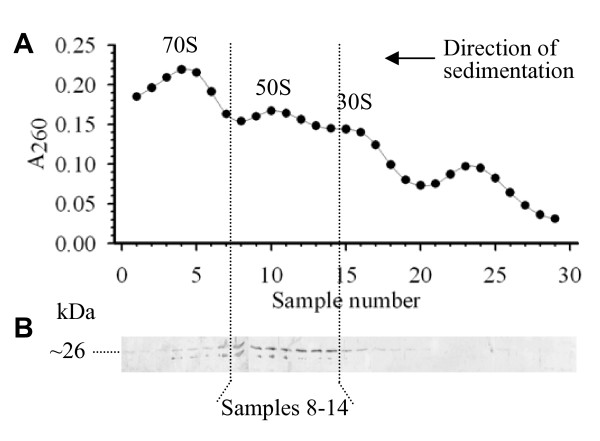
**Association of YsxC with ribosomal subunits**. A) A_260 _of a ribosome containing fraction of *S. aureus *SH1000 separated by a 10-30% (w/v) sucrose gradient centrifugation. 1 ml samples were taken and analysed for RNA content (A_260_). B) Western blot of gradient samples probed with anti-YsxC.

In order to elucidate the nature of the YsxC-ribosome association, material from *S. aureus *SH1000 containing ribosomes was separated by ultracentrifugation in a sucrose gradient. This separates the ribosome into its constituents, i.e., 30 S and 50 S subunits, as well as the whole 70 S ribosome. The association of YsxC with a particular ribosomal fraction was determined by Western blot immunodetection with anti-YsxC antibodies. As shown in Figure 5 the extract contained the three expected ribosomal fractions and YsxC was primarily located in samples 8-14 corresponding to the 50 S subunit.

### Role of YsxC in the ribosome

**Figure 6:**
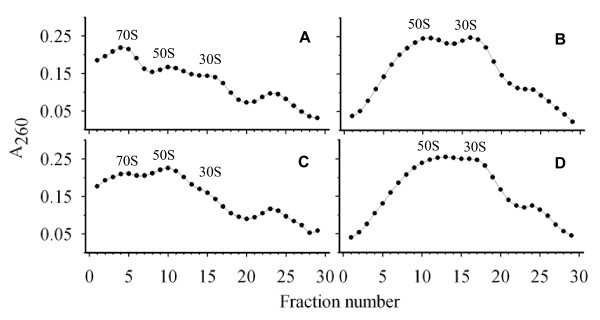
**Role of YsxC in ribosomal profile determination**. Sucrose gradient profiles were established for extracts from SH1000 (A, B) and LC109 (SH1000 P_spac_~*ysxC*/pGL485) grown with no IPTG (C, D). 10-30% (w/v) sucrose gradients were run in either associating (A, C) or dissociating (B, D) buffers and ribosomes analysed by A_260 _levels in gradient samples.

YsxC may play a role in ribosome assembly, activity or stability. Ribosome profiles of wild type and YsxC-depleted cultures were compared. Cells from both cultures were broken in the presence of two different buffers: Ribosome (associating) buffer and S (dissociating) buffer. Ribosome buffer gives conditions where tightly coupled ribosomes will remain intact whereas loosely coupled ribosomes will dissociate into subunits ([19]; Figure 6A, C). In S buffer, the magnesium levels are reduced and the monovalent ions increased which leads to full dissociation of the ribosomes ([20]; Figure 6B, D). After breakage, samples were ultracentrifuged and the pellet containing the ribosomes resuspended and loaded onto 10-30% (w/v) sucrose gradients in the relevant buffer and centrifuged. 1 ml samples were taken from the base of the gradient and tested for RNA levels (Figure 6).

The ribosome profile of the YsxC-depleted strain (LC109 grown in the absence of IPTG) in associating buffer (Figure 6C) shows a change in ratio of subunits (50 S and 30 S) to whole (70 S) ribosomes when compared to wild type (Figure 6A). The 30 S and 50 S peaks in the depleted strain were larger than that of the 70 S. In contrast, the wild type profile reveals a much larger peak for the whole ribosome than for either of the two subunits. When the ribosome is fully dissociated into its constituent subunits (in S buffer) the levels in wild type and LC109 (SH1000 P_spac_~*ysxC*/pGL485) are virtually identical (Figure 6B, D). However, the peak for the 50 S subunits is slightly broader than in the wild type potentially indicating the presence of aberrant 50 S subunits.

## Discussion

Conditional lethal constructs based on the replacement of the cognate promoters of chromosomal genes by promoters that can be exogenously controlled have been used successfully to identify essential genes in several organisms. For instance, the P_spac _promoter was used in the comprehensive genome wide study of *B. subtilis*, where *ysxC *was proven to be indispensable [6]. Identification of essential genes in *S. aureus *has also taken advantage of this system and a number of them have been identified including genes involved in cell wall biosynthesis [21,22], a glycoprotease [23] and a two-component system [24]. In this study, we have engineered the chromosomal copy of *S. aureus ysxC *under the control of P_spac_. Growth of LC109 (SH1000 P_spac_~*ysxC*/pGL485) depended on the presence of the inducer IPTG in the medium, thereby proving that *ysxC *is apparently essential in *S. aureus*. Our results are in agreement with data from an antisense study by Forsyth and co-workers suggesting the essentiality of *ysxC *in *S. aureus *[25]. In the absence of inducer, the strain is unable to form single colonies on plate and only residual growth is detected in liquid medium. The latter is most likely due to the YsxC remaining in the cell at the time of inducer removal and/or the minor leaky nature of P_spac _also observed in the P_spank _expression of *ysxC *by Schaefer and co-workers [9]. Interestingly, even maximum IPTG concentrations are unable to restore the growth rate of the mutant to the SH1000 wild type values. Thus, YsxC could potentially be an interesting target for novel drug development.

Galperin and Koonin cite YsxC in the top 10 list of 'known unknowns' of highly attractive targets for experimental study of conserved hypothetical proteins in *S. aureus *[26]. Nevertheless, it is extremely important to verify essentiality and analyse gene function in relevant pathogens as not all genes essential in one species maybe so in another.

Tandem affinity purification was originally developed in yeast [27] and has been extensively used in other organisms [28-31], however, not previously in *S. aureus*. TAP tagging of YsxC and subsequent purification indicated interactions with a number of proteins, the majority of which had functions related to or were integral parts of the ribosome. These were 30 S ribosomal proteins S2 and S10, and 50 S ribosomal protein L17. This indicates that the function of YsxC is likely to be related to the ribosome. However, the ribosome is a complex structure and a large number of processes are required for its correct function, including the construction of subunits from ribosomal proteins and RNA and the assembly of the subunits into the whole ribosome before the translation process. Much of the exact details of these processes and which additional factors are required are unknown. S2 and S10 are not located together on the assembled ribosome but involved in the later stages of 30 S assembly [32]. In contrast, 50 S ribosomal protein L17, which is localized on the surface of the subunit, binds to 23S rRNA, and even after extensive treatment to dissociate proteins can be found in the core of the 50 S subunit [33-35]. Importantly, *B. subtilis *L17 over-expression in *E. coli *results in abnormal cell division and nucleoid segregation becoming ultimately lethal [36]. Similarly, in *B. subtilis*, a mutation altering L17 was reported to cause temperature sensitivity and a sporulation defect [37]. Interestingly, depletion of YsxC in *B. subtilis *results in cell elongation, abnormal cell curvature and nucleoid condensation [38]. Similarly, depletion of YihA in *E. coli *also impairs cell division [16]. Importantly, deficiency of other small molecular weight GTPases in various species, including ObgE in *E. coli*, and Bex in *B. subtilis *also appear to affect cytokinesis and chromosome partitioning [39,40]. Whether these phenotypes are due to the absence of YsxC (and/or L17) or other P-loop GTPases directly impinging on the cell division-related apparatus or a downstream pleiotropic effect remains to be studied. Our light and transmission electron microscopy studies of the cellular morphology of *S. aureus *YsxC-depleted cultures did not reveal a conspicuous deviation from that wild type cells (data not shown).

Recent reports based on the ribosomal intermediates accumulated following YsxC depletion or Far-Western blotting analysis of purified ribosomal proteins have suggested other YsxC interacting partners in *E. coli *and/or *B. subtilis*. A few are essential for viability (L6, L7/L12, L10, L23, and perhaps L16) while others, although required for optimal growth, are dispensable (L1, L27 and L36) [9,10]. The L7/L12 stalk (which binds L10 at its base) has been suggested to participate in 23S RNA binding and on the recruitment of peripheral ribosomal factors [41]. Structural studies on the topology of several proteins including L7/12, L1, L6 and S5 has led to postulate a role for them as RNA binders probably stabilizing rRNA tertiary structure by fixing the positions of pairs of rRNA sequences [42]. The possible YsxC contribution to, RNA stabilization remains to be determined. Although the bulk of L7/L12 resides within the 50 S region, evidence of its interaction with the 30 S subunit, including S2 has been provided by cross-linking studies (See Review [43]). In addition, immuno-EM observations provide supportive evidence for different locations within the ribosome for the L7/L12 carboxy-terminal end including the 30 S subunit. It is also worth noting that most of the proteins shown to interact with YsxC are well exposed on the surface of the *E. coli *ribosome: S1 (which requires S2 for binding to the 30 S subunit), S5, L7/L12, L10, L17 [44]. Thus providing clues as to the location of YsxC within the ribosome.

Butland and co-authors found YihA (the *E. coli *YsxC homolog) to associate with itself [28]. In our study such interaction would not be detectable as only the tagged copy of the *ysxC *was present in the chromosome. However, our experimental design enabled us to confirm that the YsxC-TAP-tag protein was functional, excluding the possibility of inactive protein artefacts.

The interaction we have observed between YsxC and the β' subunit of RNA polymerase, has also been previously reported for ObgE [14,28]. Further work needs to be done to first confirm this interaction in *S. aureus *and then establish whether it relates to ribosomal or extra-ribosomal functions as reported for L24 of *B. subtilis *[45].

P-loop GTPases, such as YsxC, show an association mainly with one or other subunit of the ribosome. For instance, Era and YjeQ with the 30 S subunit [46,47], and Obg, YlqF and YphC with the 50 S subunit [9,13,48]. We have shown here that YsxC also associates with the 50 S subunit, a similar behaviour to its ortholog in *B. subtilis *[10]. Since our co-fractionation experiments revealed the interaction of YsxC with proteins from the small and large ribosome subunits, its absence from the 30 S fraction could be due to lower affinity and/or stability of YsxC towards its partners in that subunit. The specific role of YsxC and other P-loop GTPases in the assembly or stability of the 50 S subunit remains to be determined.

After YsxC depletion, the amount of 70 S ribosome decreases with concomitant subunit increase. This could be due to an error in the assembly of the subunits themselves or their assembly into the whole ribosome. As the levels of individual subunits after full dissociation stays approximately the same between wild type and YsxC depleted cells it is possible that the subunits are not being fully assembled. This was observed in *B. subtilis *where depletion of YsxC results in a number of proteins missing from the 50 S subunit, ultimately resulting in the accumulation of aberrant large subunits [10]. It has been reported by these authors that YsxC in *B. subtilis *binds the 44.5 S preribosomal particle. The depletion conditions used to enable the harvesting of sufficient biomass for ribosomal extraction required some growth of the culture, prior to cessation, which could have partially masked the presence of distinctive intermediates.

YsxC could also act at the level of ribosomal stability; once the ribosome is assembled it may require transient external proteins for stabilization, as it has been postulated for Era [49]. This could explain the interaction of ObgE, one of the P-loop GTPases, with both of the ribosomal subunits observed by Sato and co-workers in *E. coli *[14]. The dual interaction could be mediated by the presence of ribosomal constitutents modulating YsxC GTPase activity, by GTPases activating proteins (GAPs) or guanine exchange factors (GEFs) [50], or the intracellular guanine pool [51]. However, additional evidence of ObgE association with the small ribosomal particle is needed since other authors have only reported the co-fractionation of Obg homologs with the 50 S fraction in *E. coli *and other species [48,52,53].

## Conclusions

In this article we have successfully used conditional lethal genetic constructs and implemented Tandem Affinity Purification technology in *S. aureus *to show that YsxC in *S. aureus *is an apparently essential protein that associates with the large ribosomal subunit and plays a role in ribosomal assembly or ribosomal stability. Ribosomal components have been a proven target for successful antibiotics, the elucidation of the role of additional essential and highly conserved ribosomal proteins such as YsxC would open a new avenue to the discovery of novel antimicrobial drugs.

## Methods

### Media and growth conditions

Strains and plasmids are listed in Table 2. *E. coli *was grown in Luria-Bertani (LB) medium and *S. aureus *in BHI (Oxoid). Growth was carried out at 37°C, with shaking at 250 rpm for liquid media. To verify essentiality, cultures were inoculated to OD_600_~0.0001. When required, antibiotics were added at the following concentrations: ampicillin (Amp), 100 mg l^-1^; chloramphenicol (Cam), 20 mg l^-1^; erythromycin (Ery), 5 mg l^-1^; lincomycin (Lin), 25 mg l^-1^; kanamycin (Kan), 50 mg l^-1 ^and neomycin (Neo), 50 mg l^-1^; tetracycline (Tet), 5 mg l^-1^. Selection of *S. aureus *strains containing the *ery *or *kan *genes was made on Ery/Lin and Kan/Neo, respectively. The *S. aureus *NCTC8325 (SH1000 parental strain) gene homolog of the *B. subtilis ysxC *is SAOUHSC0177.

**Table 2:** Strains and plasmids used in this study

Strain	Relevant genotype/markers	Source
*Escherichia coli*		
EL250	F- *mcr *Δ(*mrr*-*hsdRMS*-*mcrBC*) ϕ 80 *lacZ *ΔM15 Δlac×74 *recA1 deoR araD139 *Δ(*ara*-*leu*) 7697 *galU galK rpsL *(StrR) *endA1 nupG *[λcl857 *araC*-PBADflpe]	[57]
GL1299	*EL250/pGL411*	This study
TunerTM(DE3) pLacI	F- *ompT hsdSB*(rB- mB-) *gal dcm lacY1 *(DE3) pLacI (Cam)	Novagen
*Staphylococcus aureus*		
LC101	*RN4220 ysxC::TAP-tag*	This study
LC102	*SH1000 spa*	This study
LC103	*SH1000 spa ysxC::TAP-tag*	This study
LC107	RN4220 P_spac _~*ysxC ysxC+*	This study
LC108	SH1000 P_spac _~*ysxC ysxC+*	This study
LC109	SH1000 P_spac _~*ysxC ysxC+*/pGL485	This study
RN4220	Restriction deficient transformation recipient	[58]
SH1000	Functional *rsbU*+ derivative of 8325-4	[63]
SJF590	8325-4 *spa*::*tet*	[62]

**Plasmid**	**Relevant genotype**	**Source**

pBS1479	CBP/Protein A tag	[27]
pDG1513	Tetracycline resistance gene (*tet*)	[55]
pELC1	pGL411 derivative with TAP-kan cassette in frame with 3' end of SH1000 *ysxC*	This study
pELC4	pETBlue-1-based *ysxC *His_6 _tag translational fusion	This study
pELC6	Tet-T-P_spac _cassette upstream *ysxC *gene in pGL411	This study
pETBlue-1 AccepTor	3'-dA overhang cloning plasmid vector for protein overexpression; ColE1 ori	Novagen
pGL400	Tet-T-P_spac _cassette	This study
pGL411	pOB derivative containing SH1000 *ysxC *and flanking regions	This study
pGL433	TAP-tag-kan cassette	This study
pGL485	pMJ8426-based *lacI *pE194ori *cat*	This study
pMAL7	Kanamycin resistance gene (*kan*)	[61]
pMJ8426	*lacI *pE194ori	[26]
pOB	Erythromycin/lincomycin resistance gene (*ery*); ColE1 ori	[54]

### Construction of *S. aureus *SH1000 containing a chromosomal single copy of *ysxC *under the control of a regulatable promoter

Oligonucleotide primers used are listed in Table 3 and a map of the final chromosomal construct is shown in Figure 1A. pELC6 was created by cloning the Tet-T-P_spac _cassette from pGL400 into a vector containing the *ysxC *gene region from *S. aureus *SH1000 (pGL411). pGL400 was constructed in a 3-way ligation reaction into the HindIII site of pOB [54] of the following PCR-amplified fragments: a) the *tet *resistance gene from plasmid pDG1513 [55] (670 bp fragment; primers: 5'GLUSh6B1 and 3'GLUSh6B); and, b) a 2236 bp fragment (primers: 5'GLUSh6A1 and 3'GLUSh6A) from pMUTIN [56] containing the t0t1t2 transcriptional terminators, the P_spac _promoter and the oid regulatory region. pGL411 is a pOB derivative containing the *S. aureus ysxC *region including 1397 bp upstream and 1354 bp downstream of this gene which was produced using primers 5'GLUSh3I and 3'GLUSh3I. The Tet-T-P_spac _cassette was amplified from pGL400 using primers 5'GLUSh16H and 3'GLUSh16H and inserted upstream of *ysxC *in pGL411 (strain *E. coli *GL1299) by λred recombination [57]. The resulting plasmid was named pELC6. Purified pELC6 was electroporated into *S. aureus *RN4220 [58] to create, by suicidal recombination, an intermediate strain (LC107) containing two copies of *ysxC*: a wild type and a P_spac_~*ysxC*. Using φ11 phage transduction [59] from LC107 into SH1000 the resident *ysxC *gene in SH1000 was replaced by a single copy of *ysxC *under the control of P_spac _by selecting for transductants resistant to tetracycline and sensitive to erythromycin. The resulting strain was named LC108 (SH1000 P_spac_~*ysxC*). Replacement was confirmed by Southern blot analysis (results not shown). A multicopy plasmid containing *lacI *was constructed (pGL485) and transduced into LC108 to generate LC109 (SH1000 P_spac_~*ysxC*/pGL485). pGL485 is a pMJ8426 [21] derivative where the tetracycline resistance gene between the *ClaI *and *SalI *sites has been replaced by the chloramphenicol acetyl transferase gene (*cat*) from pSK5630 [60]. The latter fragment was obtained by PCR amplification using primers, 5'GLUSh103A and 3'GLUSh103A.

**Table 3:** Oligonucleotide primers used in this study

Primer	Sequence (5' → 3')
5'GLUSh3I	ataaGGATCCtggcctgtttaataggatct^1^
3'GLUSh3I	ataaGGATCCaacttgtagcaggaagtggt^1^
3'GLUSh6A	taaatAAGCTTaattgtgagcggctcacaattccac^1^
5'GLUSh6A1	tattaaGCGGCCGCtcattgcttccaaggagctaaagaggtccctag^1^
3'GLUSh6B	atattAAGCTTagaaatccctttgagaatgttt^1^
5'GLUSh6B1	tattaaGCGGCCGCcggattttatgaccgatgatgaag^1^
5'GLUSh16H	*attaattcaatattattaggattaactttcattttatatcctcactt*aattgtgagcggctcacaattccac^2^
3'GLUSh16H	*ttcaaatattatataatggtagagttgaaagagaatataaaa*ttagaaatccctttgagaatgtt^2^
5'GLUSh65B	*cttacattatttttaaaatttttgtataagttttgtcgtacaaaaa*atcgatacaaattcctcg^2^
3'GLUSh65B	*ataataaacaacaacaaatatggaatttaattgaaccgtatatttca*atggaaaagagaagatgg^2^
5'GLUSh27A	aattgGGCGCGCCatggaaaagagaagatgg^1^
3'GLUSh27A	atttGCGGCCGCtcaggttgacttccccgcgg^1^
5'GLUSh27B	atttGCGGCCGCgataaacccagcgaaccattg^1^
3'GLUSh27B	atttGGCCGGCCatcgatacaaattcctcg^1^
5'GLUSh103A	taatgtATCGATaataatggtttcttagacg^1^
3'GLUSh103A	tattatGTCGACagtcggcattatctc^1^
5'elc4	atgaaagttaatcctaataatattg^3^
3'elc4	ttacaccaccaccaccaccactgaaatatacggttcaattaaattc^3^

^1 ^upper case bases indicate restriction sites engineered within the oligonucleotide

^2 ^italics indicate the fragment of the oligonucleotide designed for λ_red _recombination, whilst non-italics indicate the portion of the primer designed to amplify the insert; blackboxes indicate the location of the RBS and the START of *ysxC *in the complementary strand (5'GLUSh16H) or the 3' end of the *ysxC *sequence (3'GLUSh65B).

^3 ^for 3'-dA overhang ligation

### Construction of an in vivo YsxC-Tandem Affinity Purification (TAP) tagged construct in *S. aureus*

A plasmid containing the TAP-tag cassette (pGL433) linked to kanamycin resistance was constructed as follows. Two PCR-amplified fragments (ReadyMix ABgene) were ligated together at the *NotI *site: a) a fragment from pBS1479 [27] containing the Calmodulin Binding Protein (CBP)/Protein A tag (TAP-tag cassette) [30]; and, b) the kanamycin resistance gene from *Streptococcus faecalis *(*kan*) present in plasmid pMAL7 [61]. The resulting TAP-tag-*kan *cassette fragment was cloned in the A-overhang site of pCRII TOPO (Invitrogen) to give pGL433. The TAP-tag-*kan *cassette was PCR-amplified from pGL433 and inserted into pGL411 (in strain *E. coli *GL1299) by λ red recombination as a TAP-tag translational fusion to *ysxC *(plasmid pELC1). The resulting *ysxC*::TAP-tag-*kan *fragment was flanked by the chromosomal upstream (1397 bp) and downstream (1354 bp) regions surrounding *ysxC *present in pGL411. pELC1 was electroporated into *S. aureus *RN4220, which generated by single cross-over suicidal recombination a strain with two copies of *ysxC*, one wild type and one TAP-tagged, LC101. A strain was constructed with the Protein A-encoding gene (*spa*) deleted. *S. aureus *8325-4 *spa*::*tet *[62] was lysed with φ 11 and the *spa *mutation transduced into SH1000 to give LC102 (SH1000 *spa*::*tet*). Resolution of the two copies of *ysxC *in LC101 into only a *ysxC*::TAP-tagged copy was achieved by ρ 11-mediated transduction [59] of a LC101 lysate into LC102. Transductants resistant to kanamycin (*ysxC*::TAP-tag) and tetracycline (*spa*::*tet*) but sensitive to erythromycin (antibiotic marker linked to the wild type copy of *ysxC *in pELC1) would have only *ysxC*~TAP-tag in a *spa*-background, LC103 (SH1000 *spa*::*tet ysxC*::TAP-tag-*kan*). This strain was verified by Southern blot analysis (results not shown). Figure 1B shows the final chromosomal insertion, with the relevant DNA junction sequence.

### Tandem affinity purification

Cultures of LC103 were grown in BHI to mid-exponential phase (OD_600_~3.0), placed immediately onto ice slurry for 10 min, harvested by centrifugation (6,000 rpm, 10 min, 4°C, Jouan CR3i rotor AC50.10), frozen in liquid nitrogen and stored at -80°C. Subsequently, a cell extract was obtained from cells broken with a Braun homogeniser. The fraction containing membranes and ribosomes was isolated by centrifugation at 50,000 rpm for 2.5 h in a Beckman 70.1 Ti rotor. This fraction was subsequently purified using a method based on that previously reported by Puig *et al*. (2001) [27]. All binding and elution steps were performed in 0.8 × 4 cm Poly-prep columns (Bio-Rad). 200 μl of IgG-Sepharose bead suspension (Amersham Biosciences) was transferred into the column and the beads were washed with 10 ml IPP150 (10 mM Tris-HCl pH 8.0, 150 mM NaCl, 0.1% v/v Nonidet NP-40). 10 ml of extract in IPP150, corresponding to 2.5 l of original culture, was transferred into the column, sealed and rotated for 4 h at 4°C to allow binding of Protein A to the resin. Multiple purifications were run in parallel to increase protein yield. Elution to remove unbound protein was performed by gravity flow washing the beads three times with 10 ml IPP150 supplemented with Nonidet (NP40) at a final concentration of 1.5% (v/v). Protein A-bound complexes were excised from the resin by TEV protease cleavage, performed by addition of 1 ml of TEV cleavage buffer and 100 units of AcTEV protease (Invitrogen). The beads were rotated for 16 h at 4°C. The TEV cleavage eluate containing the protein complex CBP-YsxC -protein partners was recovered by gravity flow and 1 ml was mixed with 3 ml of Calmodulin Binding Buffer (10 mM Tris-HCl pH 8.0, 10 mM 2-mercaptoethanol, 150 mM NaCl, 1 mM magnesium acetate, 1 mM imidazole, 2 mM CaCl_2_, 0.1% v/v Nonidet NP-40) and 3 μl 1 M CaCl_2_. The resulting solution was applied to a column containing 200 μl of Calmodulin-Sepharose beads (Stratagene) that had been washed with 10 ml of Calmodulin Binding Buffer. The column was then rotated for 1 h at 4°C. Elution was performed by gravity flow and the beads washed three times with 10 ml Calmodulin Binding Buffer. The bound proteins were eluted with Calmodulin Elution Buffer (10 mM Tris-HCl pH 8.0, 10 mM 2-mercaptoethanol, 150 mM NaCl, 1 mM magnesium acetate, 1 mM imidazole, 2 mM EGTA, 0.1% v/v Nonidet NP-40) in 10×200 μl fractions. Proteins purifed as described from the equivalent of 15 l of original culture were TCA precipitated and separated using a gradient of 4-12% (w/v) SDS-PAGE and silver stained. Two independent and equivalent experiments were undertaken Distinctive bands were in-gel tryptic digested, and prepared for positive-ion MALDI mass spectra (Applied Byosystems 4700 Proteomics Analyzer), MS spectra were acquired, and the strongest peaks with a signal to noise greater than 40 were selected for CID-MS/MS analysis (Technology Facility, University of York). Mass spectral data were submitted to database searching using the MASCOT program (Matrix Science Ltd.) The Mowse scoring algorithm uses empirically determined factors to assign a statistical weight to each individual peptide match. The threshold level indicates that a match is significant if it would be expected to occur at random with a frequency of less than 5%. Therefore, individual ions with scores greater than the calculated threshold level indicate identity or extensive homology.

### Subcellular localisation

*S. aureus *SH1000 (2 l culture) was grown to an OD_600_~3 and immediately transferred to an ice slurry for 10 min. Cells were harvested (6,000 rpm, 10 min, 4°C), broken using a Braun homogeniser, and the membrane/ribosome fraction purified by ultracentrifugation at 50,000 rpm for 2.5 h in a 70.1 Ti rotor (Beckman). The resulting pellet was resuspended in 7 ml of 0.01 M Tris-HCl pH 8.2, 14 mM magnesium acetate, 60 mM potassium acetate, 1 mM DTT containing 0.5% (v/v) Triton X-100 to solubilise membranes. The ultracentrifugation step was repeated and the pellet resuspended in 6 ml of the above buffer containing 1 M NH_4_Cl. The sample was ultracentrifuged again and the resulting pellet was resuspended in 5 ml of the above buffer.

### YsxC overexpression, purification and production of antisera

A His(6)tagged version of YsxC was constructed by cloning the *ysxC *gene PCR-amplified from *S. aureus *SH1000 (using primers 5'elc4 and 3'elc4, and ReadyMix, ABgene) into the 3'-dA overhang site of the overexpression vector pETBlue-1 AccepTor vector (Novagen). The resulting plasmid (pELC4) was subsequently electroporated into *E. coli *TunerTM (DE3) pLacI (Novagen). Cells were grown in LB 50 μg ml^-1 ^Amp on a rotary shaker (250 rpm) at 37°C to an OD_600 _= 0.5. Then expression was induced by the addition of 0.5 mM IPTG and further incubation undertaken for 3 hrs. Cells were harvested by centrifugation at 5,500 rpm for 10 min (Jouan CR3i rotor AC50.10), and the pellet was stored at -20°C. The pellet was resuspended in 20 ml of Buffer C (50 mM Tris-HCl pH 8.0). Cells were disrupted by sonication (Sanyo MSE Soniprep 150; 16 micron amplitude, 2 × 20 sec treatments). Inclusion bodies were recovered by centrifugation at 10,000 rpm in a Beckman JA-20 rotor for 10 min and were subsequently washed three times via resuspension in 10 ml of buffer C, 10 ml buffer C plus 1 M NaCl, and 10 ml buffer C, and centrifugation. Each time pellets were suspended in the buffer and then collected by centrifugation at 10,000 rpm for 5 min (Beckman JA-20 rotor). Washed inclusion bodies were suspended in 20 ml of buffer C plus 8 M Urea, left to dissolve for 20 min with stirring and then remaining insoluble material was removed by centrifugation in a Beckman JA-20 rotor at 19,000 rpm for 15 min at 4°C. The sample was applied on a 12 ml Ni-column (iminodiacetic acid as a chelator immobilized on Sepharose 6B FF, Sigma). The column was washed with 25 ml of 8 M Urea in buffer C, then with 25 ml 8 M urea in 50 mM 2-(N-morpholino)ethane sulphonic acid (MES)/NaOH buffer pH 6.3 and finally with 25 ml of 8 M Urea in 50 mM sodium acetate buffer pH 4.6. The pH 6.3 wash contained the recombinant protein and was concentrated using a VivaSpin concentrator 100000 MWCO (Viva Science). Samples were applied on a Hi-Load Superdex 200 16 × 60 cm (Amersham) equilibrated with 6 M Urea in buffer C. Proteins were eluted from the column in the same buffer and 2 ml fractions were collected and analysed for protein content. The resulting protein was dialysed against PBS. 1 mg of the purified protein was then used for production of polyclonal antibodies against YsxC (Antibody Resource Centre, University of Sheffield).

### Sucrose gradient centrifugation

SH1000 and LC109 (SH1000 P_spac_~*ysxC*/pGL485) inoculated to an starting OD600~0.01 and grown to an OD_600_~0.5 in BHI and BHI plus 20 μg ml^-1 ^Cam, respectively. Growth of LC109 in the absence of IPTG results in noticeable but partial YsxC depletion. After breakage with a Braun homogeniser, cell extracts were centrifuged at 50,000 rpm for 2.5 h in a Beckman 70.1 Ti rotor at 4°C. The supernatant was removed and the pellet resuspended in 2 ml of either S buffer [20] or Ribosome buffer [19]. Both buffers were supplemented with protease inhibitors (Complete, Roche; 1 tablet in 25 ml and added at a 1:25 dilution to the reaction mixture). 30 ml 10-30% (w/v) sucrose gradients were formed using a Hoefer gradient maker. Samples corresponding to 2 l of original culture were layered on top of the gradient and centrifuged at 19,000 rpm for 16 h at 4°C in a Beckman SW28 rotor. 1 ml fractions were removed from the base of the gradient and RNA levels were assayed by diluting each sample 1:100 in Tris-HCl pH 7.8 and measuring absorbance at 260 nm.

## List of Abbreviations

TAP: tandem affinity purification; CBP: calmodulin binding protein.

## Authors' contributions

ELC, JGL and SJF contributed in the design of the study and in the writing of the manuscript. ELC and JGL carried out the genetic constructs necessary for the work and the determinations of *ysxC *essentiality. ELC performed the purification of YsxC partners, its subcellular localization, and its association with the ribosome. All authors read and approved manuscript.

## References

[B1] Shopsin B Mathema B Martinez J Ha E Campo ML Fierman A Krasinski K Kornblum J Alcabes P Waddington M Prevalence of methicillin-resistant and methicillin-susceptible *Staphylococcus aureus *in the community J Infect Dis 2000 182 359 362 10.1086/315695 10882625

[B2] National Nosocomial Infections Surveillance (NNIS) System Report, data summary from January 1992 through June 2004, issued October 2004 Am J Infect Control 2004 32 470 485 10.1016/j.ajic.2004.10.001 15573054

[B3] Tiemersma EW Bronzwaer SL Lyytikainen O Degener JE Schrijnemakers P Bruinsma N Monen J Witte W Grundman H Methicillin-resistant *Staphylococcus aureus *in Europe, 1999-2002 Emerg Infect Dis 2004 10 1627 1634 15498166 10.3201/eid1009.040069 PMC3320277

[B4] Zetola N Francis JS Nuermberger EL Bishai WR Community-acquired meticillin-resistant *Staphylococcus aureus*: an emerging threat Lancet Infect Dis 2005 5 275 286 10.1016/S1473-3099(05)70112-2 15854883

[B5] Hutchison CA Peterson SN Gill SR Cline RT White O Fraser CM Smith HO Venter JC Global transposon mutagenesis and a minimal *Mycoplasma *genome Science 1999 286 2165 2169 10.1126/science.286.5447.2165 10591650

[B6] Kobayashi K Ehrlich SD Albertini A Amati G Andersen KK Arnaud M Asai K Ashikaga S Aymerich S Bessieres P Essential *Bacillus subtilis *genes Proc Natl Acad Sci USA 2003 100 4678 4683 10.1073/pnas.0730515100 12682299 PMC153615

[B7] Caldon CE March PE Function of the universally conserved bacterial GTPases Curr Opin Microbiol 2003 6 135 139 10.1016/S1369-5274(03)00037-7 12732302

[B8] Comartin DJ Brown ED Non-ribosomal factors in ribosome subunit assembly are emerging targets for new antibacterial drugs Curr Opin Pharmacol 2006 6 453 458 10.1016/j.coph.2006.05.005 16890019

[B9] Schaefer L Uicker WC Wicker-Planquart C Foucher AE Jault JM Britton RA Multiple GTPases participate in the assembly of the large ribosomal subunit in *Bacillus subtilis* J Bacteriol 2006 188 8252 8258 10.1128/JB.01213-06 16997968 PMC1698177

[B10] Wicker-Planquart C Foucher AE Louwagie M Britton RA Jault JM Interactions of an essential *Bacillus subtilis *GTPase, YsxC, with ribosomes J Bacteriol 2007 190 681 690 10.1128/JB.01193-07 17981968 PMC2223697

[B11] Campbell TL Daigle DM Brown ED Characterization of the *Bacillus subtilis *GTPase YloQ and its role in ribosome function Biochem J 2005 389 843 852 10.1042/BJ20050733 15828870 PMC1180735

[B12] Datta K Skidmore JM Pu K Maddock JR The *Caulobacter crescentus *GTPase CgtAC is required for progression through the cell cycle and for maintaining 50 S ribosomal subunit levels Mol Microbiol 2004 54 1379 1392 10.1111/j.1365-2958.2004.04354.x 15554976

[B13] Matsuo Y Morimoto T Kuwano M Loh PC Oshima T Ogasawara N The GTP-binding protein YlqF participates in the late step of 50 S ribosomal subunit assembly in *Bacillus subtilis* J Biol Chem 2006 281 8110 8117 10.1074/jbc.M512556200 16431913

[B14] Sato A Kobayashi G Hayashi H Yoshida H Wada A Maeda M Hiraga S Takeyasu K Wada C The GTP binding protein Obg homolog ObgE is involved in ribosome maturation Genes Cells 2005 10 393 408 10.1111/j.1365-2443.2005.00851.x 15836769

[B15] Uicker WC Schaefer L Koenigsknecht M Britton RA The essential GTPase YqeH is required for proper ribosome assembly in *Bacillus subtilis* J Bacteriol 2007 189 2926 2929 10.1128/JB.01654-06 17237168 PMC1855813

[B16] Dassain M Leroy A Colosetti L Carole S Bouche JP A new essential gene of the 'minimal genome' affecting cell division Biochimie 1999 81 889 895 10.1016/S0300-9084(99)00207-2 10572302

[B17] Pragai Z Harwood CR YsxC, a putative GTP-binding protein essential for growth of *Bacillus subtilis *168 J Bacteriol 2000 182 6819 6823 10.1128/JB.182.23.6819-6823.2000 11073929 PMC111427

[B18] Ruzheinikov SN Das SK Sedelnikova SE Baker PJ Artymiuk PJ Garcia-Lara J Foster SJ Rice DW Analysis of the open and closed conformations of the GTP-binding protein YsxC from *Bacillus subtilis* J Mol Biol 2004 339 265 278 10.1016/j.jmb.2004.03.043 15136032

[B19] Blaha G Stelzl U Spahn CM Agrawal RK Frank J Nierhaus KH Preparation of functional ribosomal complexes and effect of buffer conditions on tRNA positions observed by cryoelectron microscopy Methods Enzymol 2000 317 292 309 full_text 10829287 10.1016/s0076-6879(00)17021-1

[B20] Champney WS Burdine R Macrolide antibiotics inhibit 50 S ribosomal subunit assembly in *Bacillus subtilis *and *Staphylococcus aureus* Antimicrob Agents Chemother 1995 39 2141 2144 8540733 10.1128/aac.39.9.2141 PMC162898

[B21] Jana M Luong TT Komatsuzawa H Shigeta M Lee CY A method for demonstrating gene essentiality in *Staphylococcus aureus* Plasmid 2000 44 100 104 10.1006/plas.2000.1473 10873532

[B22] Sobral RG Ludovice AM de Lencastre H Tomasz A Role of *murF *in cell wall biosynthesis: isolation and characterization of a *murF *conditional mutant of *Staphylococcus aureus* J Bacteriol 2006 188 2543 2553 10.1128/JB.188.7.2543-2553.2006 16547042 PMC1428427

[B23] Zheng L Yang J Landwehr C Fan F Ji Y Identification of an essential glycoprotease in *Staphylococcus aureus* FEMS Microbiol Lett 2005 245 279 285 10.1016/j.femsle.2005.03.017 15837383

[B24] Dubrac S Msadek T Identification of genes controlled by the essential YycG/YycF two-component system of *Staphylococcus aureus* J Bacteriol 2004 186 1175 1181 10.1128/JB.186.4.1175-1181.2004 14762013 PMC344212

[B25] Forsyth RA Haselbeck RJ Ohlsen KL Yamamoto RT Xu H Trawick JD Wall D Wang L Brown-Driver V Froelich JM A genome-wide strategy for the identification of essential genes in *Staphylococcus aureus* Mol Microbiol 2002 43 1387 1400 10.1046/j.1365-2958.2002.02832.x 11952893

[B26] Galperin MY Koonin EV 'Conserved hypothetical' proteins: prioritization of targets for experimental study Nucleic Acids Res 2004 32 5452 5463 10.1093/nar/gkh885 15479782 PMC524295

[B27] Puig O Caspary F Rigaut G Rutz B Bouveret E Bragado-Nilsson E Wilm M Seraphin B The tandem affinity purification (TAP) method: a general procedure of protein complex purification Methods 2001 24 218 229 10.1006/meth.2001.1183 11403571

[B28] Butland G Peregrin-Alvarez JM Li J Yang W Yang X Canadien V Starostine A Richards D Beattie B Krogan N Interaction network containing conserved and essential protein complexes in *Escherichia coli* Nature 2005 433 531 537 10.1038/nature03239 15690043

[B29] Estevez AM Kempf T Clayton C The exosome of *Trypanosoma brucei* Embo J 2001 20 3831 3839 10.1093/emboj/20.14.3831 11447124 PMC125547

[B30] Rohila JS Chen M Cerny R Fromm ME Improved tandem affinity purification tag and methods for isolation of protein heterocomplexes from plants Plant J 2004 38 172 181 10.1111/j.1365-313X.2004.02031.x 15053770

[B31] Westermarck J Weiss C Saffrich R Kast J Musti AM Wessely M Ansorge W Seraphin B Wilm M Valdez BC Bohmann D The DEXD/H-box RNA helicase RHII/Gu is a co-factor for c-Jun-activated transcription Embo J 2002 21 451 460 10.1093/emboj/21.3.451 11823437 PMC125820

[B32] Held WA Ballou B Mizushima S Nomura M Assembly mapping of 30 S ribosomal proteins from *Escherichia coli*. Further studies J Biol Chem 1974 249 3103 3111 4598121

[B33] Homann HE Nierhaus KH Ribosomal proteins. Protein compositions of biosynthetic precursors and artifical subparticles from ribosomal subunits in *Escherichia coli *K 12 Eur J Biochem 1971 20 249 257 10.1111/j.1432-1033.1971.tb01388.x 4934681

[B34] Marquardt O Roth HE Wystup G Nierhaus KH Binding of *Escherichia coli *ribosomal proteins to 23 S RNA under reconstitution conditions for the 50 S subunit Nucleic Acids Res 1979 6 3641 3650 10.1093/nar/6.11.3641 386275 PMC327962

[B35] Stoffler-Meilicke M Noah M Stoffler G Location of eight ribosomal proteins on the surface of the 50 S subunit from *Escherichia coli* Proc Natl Acad Sci USA 1983 80 6780 6784 10.1073/pnas.80.22.6780 6359156 PMC390069

[B36] Zouine M Beloin C Ghelis C Le Hegarat F The L17 ribosomal protein of *Bacillus subtilis *binds preferentially to curved DNA Biochimie 2000 82 85 91 10.1016/S0300-9084(00)00184-X 10717392

[B37] Sharrock RA Leighton T Intergenic suppressors of temperature-sensitive sporulation in *Bacillus subtilis *are allele non-specific Mol Gen Genet 1981 183 532 537 10.1007/BF00268777 6801427

[B38] Morimoto T Loh PC Hirai T Asai K Kobayashi K Moriya S Ogasawara N Six GTP-binding proteins of the Era/Obg family are essential for cell growth in *Bacillus subtilis* Microbiology 2002 148 3539 3552 12427945 10.1099/00221287-148-11-3539

[B39] Kobayashi G Moriya S Wada C Deficiency of essential GTP-binding protein ObgE in *Escherichia coli *inhibits chromosome partition Mol Microbiol 2001 41 1037 1051 10.1046/j.1365-2958.2001.02574.x 11555285

[B40] Minkovsky N Zarimani A Chary VK Johnstone BH Powell BS Torrance PD Court DL Simons RW Piggot PJ Bex, the *Bacillus subtilis *homolog of the essential *Escherichia coli *GTPase Era, is required for normal cell division and spore formation J Bacteriol 2002 184 6389 6394 10.1128/JB.184.22.6389-6394.2002 12399511 PMC151948

[B41] Diaconu M Kothe U Schlunzen F Fischer N Harms JM Tonevitsky AG Stark H Rodnina MV Wahl MC Structural basis for the function of the ribosomal L7/12 stalk in factor binding and GTPase activation Cell 2005 121 991 1004 10.1016/j.cell.2005.04.015 15989950

[B42] Moore PB The three-dimensional structure of the ribosome and its components Annu Rev Biophys Biomol Struct 1998 27 35 58 10.1146/annurev.biophys.27.1.35 9646861

[B43] Chandra Sanyal S Liljas A The end of the beginning: structural studies of ribosomal proteins Curr Opin Struct Biol 2000 10 633 636 10.1016/S0959-440X(00)00143-3 11114498

[B44] Agafonov DE Kolb VA Spirin AS Proteins on ribosome surface: measurements of protein exposure by hot tritium bombardment technique Proc Natl Acad Sci USA 1997 94 12892 12897 10.1073/pnas.94.24.12892 9371771 PMC24234

[B45] Zouine M Beloin C Deneubourg AM Hirschbein L Le Hegarat F Overproduction, purification and characterization of the HPB12-L24 ribosomal protein of *Bacillus subtilis* FEMS Microbiol Lett 1996 145 41 48 10.1111/j.1574-6968.1996.tb08554.x 8931325

[B46] Daigle DM Brown ED Studies of the interaction of *Escherichia coli *YjeQ with the ribosome *in vitro* J Bacteriol 2004 186 1381 1387 10.1128/JB.186.5.1381-1387.2004 14973029 PMC344419

[B47] Sayed A Matsuyama S Inouye M Era, an essential *Escherichia coli *small G-protein, binds to the 30 S ribosomal subunit Biochem Biophys Res Commun 1999 264 51 54 10.1006/bbrc.1999.1471 10527840

[B48] Scott JM Ju J Mitchell T Haldenwang WG The *Bacillus subtilis *GTP binding protein *obg *and regulators of the sigma(B) stress response transcription factor cofractionate with ribosomes J Bacteriol 2000 182 2771 2777 10.1128/JB.182.10.2771-2777.2000 10781545 PMC101985

[B49] Sharma MR Barat C Wilson DN Booth TM Kawazoe M Hori-Takemoto C Shirouzu M Yokoyama S Fucini P Agrawal RK Interaction of Era with the 30 S ribosomal subunit implications for 30 S subunit assembly Mol Cell 2005 18 319 329 10.1016/j.molcel.2005.03.028 15866174

[B50] Trahey M McCormick F A cytoplasmic protein stimulates normal N-ras p21 GTPase, but does not affect oncogenic mutants Science 1987 238 542 545 10.1126/science.2821624 2821624

[B51] Lin B Covalle KL Maddock JR The *Caulobacter crescentus *CgtA protein displays unusual guanine nucleotide binding and exchange properties J Bacteriol 1999 181 5825 5832 10482526 10.1128/jb.181.18.5825-5832.1999 PMC94105

[B52] Jiang M Datta K Walker A Strahler J Bagamasbad P Andrews PC Maddock JR The *Escherichia coli *GTPase CgtAE is involved in late steps of large ribosome assembly J Bacteriol 2006 188 6757 6770 10.1128/JB.00444-06 16980477 PMC1595513

[B53] Sikora AE Zielke R Datta K Maddock JR The *Vibrio harveyi *GTPase CgtAV is essential and is associated with the 50 S ribosomal subunit J Bacteriol 2006 188 1205 1210 10.1128/JB.188.3.1205-1210.2006 16428430 PMC1347350

[B54] Horsburgh MJ Wharton SJ Cox AG Ingham E Peacock S Foster SJ MntR modulates expression of the PerR regulon and superoxide resistance in *Staphylococcus aureus *through control of manganese uptake Mol Microbiol 2002 44 1269 1286 10.1046/j.1365-2958.2002.02944.x 12028379

[B55] Guerout-Fleury AM Shazand K Frandsen N Stragier P Antibiotic-resistance cassettes for *Bacillus subtilis* Gene 1995 167 335 336 10.1016/0378-1119(95)00652-4 8566804

[B56] Vagner V Dervyn E Ehrlich SD A vector for systematic gene inactivation in *Bacillus subtilis* Microbiology 1998 144 Pt 11 3097 3104 10.1099/00221287-144-11-3097 9846745

[B57] Lee EC Yu D Martinez de Velasco J Tessarollo L Swing DA Court DL Jenkins NA Copeland NG A highly efficient *Escherichia coli*-based chromosome engineering system adapted for recombinogenic targeting and subcloning of BAC DNA Genomics 2001 73 56 65 10.1006/geno.2000.6451 11352566

[B58] Kreiswirth BN Lofdahl S Betley MJ O'Reilly M Schlievert PM Bergdoll MS Novick RP The toxic shock syndrome exotoxin structural gene is not detectably transmitted by a prophage Nature 1983 305 709 712 10.1038/305709a0 6226876

[B59] Novick RP Genetic systems in staphylococci Methods Enzymol 1991 204 587 636 full_text 1658572 10.1016/0076-6879(91)04029-n

[B60] Grkovic S Brown MH Hardie KM Firth N Skurray RA Stable low-copy-number *Staphylococcus aureus *shuttle vectors Microbiology 2003 149 785 794 10.1099/mic.0.25951-0 12634346

[B61] Horsburgh MJ Clements MO Crossley H Ingham E Foster SJ PerR controls oxidative stress resistance and iron storage proteins and is required for virulence in *Staphylococcus aureus* Infect Immun 2001 69 3744 3754 10.1128/IAI.69.6.3744-3754.2001 11349039 PMC98383

[B62] Hartleib J Kohler N Dickinson RB Chhatwal GS Sixma JJ Hartford OM Foster TJ Peters G Kehrel BE Herrmann M Protein A is the von Willebrand factor binding protein on *Staphylococcus aureus* Blood 2000 96 2149 2156 10979960

[B63] Horsburgh MJ Aish JL White IJ Shaw L Lithgow JK Foster SJ sigmaB modulates virulence determinant expression and stress resistance: characterization of a functional rsbU strain derived from *Staphylococcus aureus *8325-4 J Bacteriol 2002 184 5457 5467 10.1128/JB.184.19.5457-5467.2002 12218034 PMC135357

